# Validated Pretreatment Prediction Models for Response to Neoadjuvant Therapy in Patients with Rectal Cancer: A Systematic Review and Critical Appraisal

**DOI:** 10.3390/cancers15153945

**Published:** 2023-08-03

**Authors:** Max D. Tanaka, Barbara M. Geubels, Brechtje A. Grotenhuis, Corrie A. M. Marijnen, Femke P. Peters, Stevie van der Mierden, Monique Maas, Alice M. Couwenberg

**Affiliations:** 1 Department of Radiation Oncology, The Netherlands Cancer Institute, 1066 CX Amsterdam, The Netherlands; 2 Department of Surgery, The Netherlands Cancer Institute, 1066 CX Amsterdam, The Netherlands; 3 Department of Surgery, Catharina Hospital, 5602 ZA Eindhoven, The Netherlands; 4 GROW School for Oncology and Reproduction, Maastricht University, 6200 MD Maastricht, The Netherlands; 5 Department of Radiation Oncology, Leiden University Medical Centre, 2333 ZA Leiden, The Netherlands; 6 Scientific Information Service, The Netherlands Cancer Institute, 1066 CX Amsterdam, The Netherlands; 7 Department of Radiology, The Netherlands Cancer Institute, 1066 CX Amsterdam, The Netherlands

**Keywords:** rectal cancer, prediction models, response, neoadjuvant therapy, organ preservation, PROBAST

## Abstract

**Simple Summary:**

Organ preservation strategies can be offered to patients with rectal cancer that show a strong response to preoperative treatment in order to avoid major surgery. Prediction models may help identify these patients before the start of preoperative treatment, when treatment can still be adapted. We systematically reviewed validated pretreatment prediction models for response to preoperative treatment in patients with rectal cancer. Sixteen studies were included in this review. All studies were considered to have a high risk of bias and external validation was missing. Nevertheless, some studies show promising results, which could serve as a foundation for future research. Our systematic review provides a comprehensive overview of the current state of the literature regarding pretreatment prediction models for response to preoperative treatment in patients with rectal cancer.

**Abstract:**

Pretreatment response prediction is crucial to select those patients with rectal cancer who will benefit from organ preservation strategies following (intensified) neoadjuvant therapy and to avoid unnecessary toxicity in those who will not. The combination of individual predictors in multivariable prediction models might improve predictive accuracy. The aim of this systematic review was to summarize and critically appraise validated pretreatment prediction models (other than radiomics-based models or image-based deep learning models) for response to neoadjuvant therapy in patients with rectal cancer and provide evidence-based recommendations for future research. MEDLINE via Ovid, Embase.com, and Scopus were searched for eligible studies published up to November 2022. A total of 5006 studies were screened and 16 were included for data extraction and risk of bias assessment using Prediction model Risk Of Bias Assessment Tool (PROBAST). All selected models were unique and grouped into five predictor categories: clinical, combined, genetics, metabolites, and pathology. Studies generally included patients with intermediate or advanced tumor stages who were treated with neoadjuvant chemoradiotherapy. Evaluated outcomes were pathological complete response and pathological tumor response. All studies were considered to have a high risk of bias and none of the models were externally validated in an independent study. Discriminative performances, estimated with the area under the curve (AUC), ranged per predictor category from 0.60 to 0.70 (clinical), 0.78 to 0.81 (combined), 0.66 to 0.91 (genetics), 0.54 to 0.80 (metabolites), and 0.71 to 0.91 (pathology). Model calibration outcomes were reported in five studies. Two collagen feature-based models showed the best predictive performance (AUCs 0.83–0.91 and good calibration). In conclusion, some pretreatment models for response prediction in rectal cancer show encouraging predictive potential but, given the high risk of bias in these studies, their value should be evaluated in future, well-designed studies.

## 1. Introduction

Due to optimization of rectal cancer diagnosis and treatment, most notably by the introduction of total mesorectal excision (TME) and neoadjuvant therapy, local recurrence rates have been significantly reduced [1,2]. Neoadjuvant therapy is usually given for more advanced tumors, as either long-course chemoradiotherapy (CRT) or short-course radiotherapy (SCRT), to obtain downsizing of the tumor. More recently, total neoadjuvant treatment (TNT) has emerged as an alternative to decrease the number of distant metastases without compromising local control [2,3,4,5]. The positive effect of neoadjuvant therapy followed by TME on oncological outcomes has been widely recognized, although it is also associated with significant acute and long-term gastro-intestinal, urinary, and sexual morbidity [6,7].

A selected group of patients with rectal cancer shows no residual viable tumor cells in the resected specimen after neoadjuvant therapy, known as a pathological complete response (pCR) [8]. Preoperatively, a clinical complete response (cCR) can be defined with high accuracy based on digital rectal examination, rectoscopy, and MRI [9]. Patients with a cCR can be offered a “watch and wait” (W&W) strategy to avoid major surgery. Some patients with a near-cCR (ncCR) can be treated with additional treatment to avoid TME, such as local excision or contact X-ray brachytherapy (Papillion) [10,11,12], followed by a W&W approach if a complete response is achieved. Accumulating evidence shows that a W&W strategy might be considered as a safe alternative to TME surgery [6,13,14,15]. Furthermore, the W&W strategy is associated with favorable functional outcomes, good quality of life [16,17], and avoids a permanent stoma in patients with a very low tumor [18,19]. For some patients, the W&W strategy may be the optimal treatment option, and carefully comparing the advantages and challenges of this strategy versus standard TME should be part of a shared decision-making process.

The chance of achieving a complete response after neoadjuvant therapy is partly dependent on the neoadjuvant treatment regimen. In patients with locally advanced rectal cancer (LARC), CRT results in pCR rates of 10–30% [3], whereas TNT increases this chance [2,3,4,5], with better outcomes after the use of consolidation therapy versus induction therapy [20,21]. Nevertheless, TNT is associated with a higher risk of toxicity [20], which may impair patients’ quality of life. Careful selection of patients for type of neoadjuvant regimen is therefore essential.

Eligibility for a W&W strategy is assessed after completion of neoadjuvant therapy. Response assessment is generally recommended 12 weeks after the start of neoadjuvant CRT or SCRT and, in case of a ncCR, again at 16–20 weeks [22]. For TNT, timing of response assessment depends on the duration of the treatment and could take up to 38 weeks [22]. Ideally, selection of eligible patients for a W&W strategy is performed before the start of neoadjuvant therapy. Pretreatment response prediction would allow for personalized neoadjuvant approaches based on individual patient characteristics. This is particularly important now that neoadjuvant therapy is intensified and more often used in patients with early tumor stages, with the intention of achieving a higher rate of organ preservation [23,24,25,26,27,28,29]. In order to select patients who will benefit the most from (intensified) neoadjuvant therapy and avoid unnecessary toxicity in those who are ineligible for W&W but still require TME surgery, pretreatment response prediction is critical.

However, pretreatment prediction of response is highly challenging, as rectal cancer is a heterogeneous disease and response to neoadjuvant therapy can vary greatly, indicating a complex relationship between tumor characteristics and treatment response [30,31]. As a result, no individual pretreatment predictor currently has the ability to accurately select all eligible patients for organ preservation [31]. To improve predictive accuracy, individual pretreatment predictors can be combined in multivariable prediction models. Recently, several publications have shown the predictive potential of radiomics-based models (which extract numerous quantitative features from medical images [32]) and imaged-based deep learning models [32,33,34]. Other promising candidate predictors for pretreatment models include conventional clinical factors that are commonly measured during routine diagnostic work-up, like cTNM-stage and carcinoembryonic antigen (CEA) [31,35], or more novel predictors such as mismatch-repair (MMR) status now that immunotherapy shows encouraging results in MMR-deficient tumors [21]. At the moment, an overview of pretreatment prediction models that combine predictors other than image-based features is lacking.

In this systematic review, we aim to summarize and critically appraise validated pretreatment prediction models, other than radiomics-based models or image-based deep learning models, for response to neoadjuvant therapy in patients with rectal cancer and provide evidence-based recommendations for future research.

## 2. Materials and Methods

### 2.1. Reporting

This systematic review is reported according to the “Preferred Reporting Items for Systematic reviews and Meta-Analyses” (PRISMA) statement [36] and the PRISMA extension for searching (PRISMA-S) [37]. The research protocol was registered in PROSPERO (CRD42023385057).

### 2.2. Search Strategy

MEDLINE via Ovid, Embase.com, and Scopus were searched by an information specialist (SvdM). The schematic search was as follows: Rectal Cancer AND Response AND (Neoadjuvant therapy OR CRT/SCRT/TNT). Free text terms including synonyms were used. In addition, thesaurus terms were used for MEDLINE (MeSH) and Embase (Emtree). See Supplementary Table S1 for the complete search. No filters, restrictions, or limits were used, except for the exclusion of references published prior to 1 January 2012. The search was not based on previous work and reviewed by a second information specialist. It was originally executed on 9 June 2022 and updated on 18 November 2022. All searches were performed separately on their respective platforms (i.e., no federated searches). The results were deduplicated in EndNote 20 [38], first based on PMID, second on DOI, and finally a manual check was performed. Forward and backward citation chasing (“snowballing”) was performed for the included articles. No other databases, registries, or alternative methods were used.

### 2.3. Eligibility Criteria

#### 2.3.1. General Inclusion and Exclusion Criteria

Studies investigating a multivariable (at least two predictors) pretreatment prediction model for response to neoadjuvant therapy were selected if they met the following criteria: (1) rectal cancer patients without metastases (cT1-4N0-2M0), (2) adenocarcinoma, (3) neoadjuvant radiotherapy with or without additional systemic therapy, (4) outcome defined as either pCR, pathological tumor response, cCR, or ncCR followed by additional successful organ-preserving treatment, (5) at least one development or validation cohort with a sample size of 50 or more, and (6) any form of internal or external validation. The following study types were excluded: studies that included patients with mucinous, signet-ring cell or neuroendocrine tumors, studies including radiomics-based models or image-based deep learning models, non-English studies, studies published in non-peer-reviewed journals, conference abstracts, case reports, preclinical studies, (systematic) reviews, and meta-analyses.

#### 2.3.2. Development and Validation Studies

Studies with type 1b-4 models, according to the “transparent reporting of a multivariable prediction model for individual prognosis or diagnosis” (TRIPOD) statement, were selected (Table 1) [39]. Type 1a studies do not include any form of validation and were excluded from this review. Type 1b-2 studies use a single dataset for model development and validation. Type 1b and 2a are internal validation studies. Type 1b studies use resampling methods (e.g., cross-validation or bootstrapping) and type 2a studies randomly split the data into a development and validation cohort. Type 2b studies nonrandomly split the data into a development and validation cohort, for instance, based on location (e.g., different hospital) or time (different moment of inclusion). Because of the nonrandom variation, type 2b can be seen as intermediary between internal and external validation, according to TRIPOD [39]. Type 3 and 4 are external validation studies. Type 3 studies use separate development and validation cohorts; type 4 studies do not develop a model but only validate an existing one.

### 2.4. Article Selection and Data Extraction

Using the eligibility criteria of Section 2.3, two independent reviewers (MDT and BMG) screened titles and abstracts, using Rayyan [40], to identify potential articles. The same independent reviewers performed subsequent full-text eligibility assessment. Reasons for exclusion of ineligible articles were recorded. Included articles had to be approved by both reviewers. Disagreements were initially resolved by discussion between the reviewers and, if necessary, with help from a third reviewer (AMC). The data extraction form was based on the TRIPOD statement [39], the 11 domains of the “Critical Appraisal and Data Extraction for Systematic Reviews of Prediction Modelling Studies” (CHARMS) checklist [41], and the “Prediction model Risk Of Bias Assessment Tool” (PROBAST) [42]. Completed data extraction forms of the first five articles were discussed by MDT, BMG, and AMC to ensure that no relevant data were missed. Data extraction of the remaining articles was performed by MDT. Authors were not contacted in case of missing data. The data extraction form and extracted data have not been made publicly available.

### 2.5. Risk of Bias Assessment

PROBAST was used for risk of bias (ROB) assessment and evaluation of concerns regarding the applicability of a model [42,43]. ROB is assessed across four domains: participants, predictors, outcome, and analysis. Applicability is assessed across the first three domains. Each domain contains specific signaling questions to help with the final ROB and applicability rating; some questions are only related to model development studies. As suggested by PROBAST, a prediction model was considered to have high risk of bias or high concerns regarding applicability if at least one domain was judged as high. An unclear rating was given if at least one domain was judged as unclear and none of the other domains were judged as high. Consequently, a prediction model was only judged as low risk if all domains were graded as such. PROBAST forms of included articles were completed by MDT and discussed by MDT, BMG, and AMC.

### 2.6. Data Synthesis

For each prediction model, relevant information and ROB assessment was summarized in a descriptive synthesis, supported by tables and figures. Predictive performance measures that were reported included model discrimination and calibration. Discrimination is often quantified and reported with the area under the curve (AUC). The apparent AUC (discrimination of the model on the development cohort before internal validation) and validated AUCs were reported. An AUC of 1.0 indicates perfect discrimination. As a general rule of thumb, AUC values can be considered as poor (0.5–0.7), moderate (0.7–0.8), good (0.8–0.9), or excellent (0.9–1.0) [44]. Calibration is commonly evaluated with a goodness-of-fit test, such as the Hosmer–Lemeshow test (a *p*-value < 0.05 indicates miscalibration [45]), or graphically with a calibration curve, which can be quantified by the calibration slope and intercept. If a study included multiple prediction models, results of the model with the best discrimination were reported. Prediction models were categorized according to type of included predictors. No meta-analysis was performed due to expected heterogeneity.

## 3. Results

### 3.1. Search

**Figure 1:**
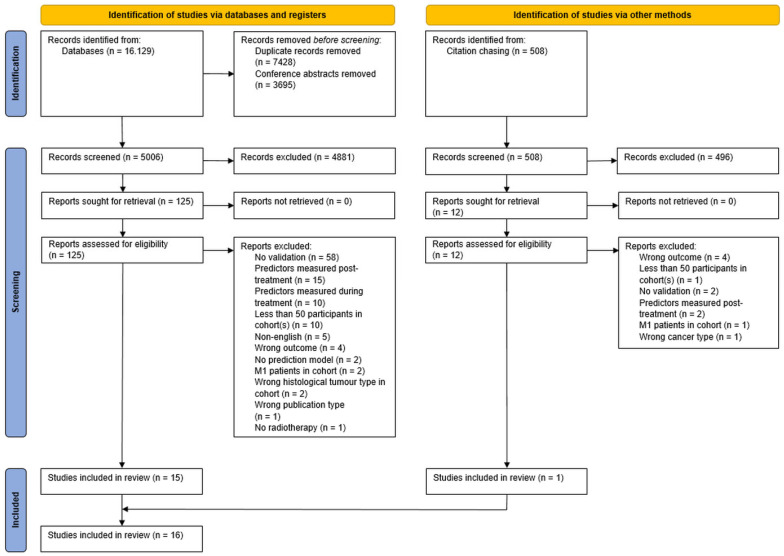
PRISMA flow diagram of systematic review.

In total, 16.129 studies were identified by our literature search. After removal of conference abstracts and deduplication, titles and abstracts of 5006 studies were screened. In total, 4881 studies were excluded, and 125 studies were assessed for eligibility through full text review, resulting in the inclusion of 15 studies. Forward and backward citation chasing of included studies identified one additional study [46]. Sixteen studies were included for data extraction and ROB assessment [46,47,48,49,50,51,52,53,54,55,56,57,58,59,60,61]. See the PRISMA flow diagram (Figure 1) for an overview of the search process.

### 3.2. Overview of Study Populations

Table 2 shows the cohort characteristics of selected studies. Studies were published between 2014 and 2022, 14 in the last five years [46,48,49,51,52,53,54,55,56,57,58,59,60,61]. Patients were treated between 1998 and 2021. Sample sizes of development and validation cohorts ranged from 14 to 666 participants. Four studies consisted of at least one prospective cohort [46,50,52,55], four studies consisted of one or more multicenter cohorts [52,56,57,58], while nine studies were single-center retrospective cohort studies [47,48,49,51,53,54,59,60,61]. Tumor stages were generally intermediate or advanced (TNM stage cT3-4N0-2/stage II-III) and the neoadjuvant treatment regimen was CRT in all studies. In one study, CRT was preceded by induction chemotherapy [59] and, in three studies, CRT was followed by consolidation chemotherapy [46,55,60]. In all but one study, a standard total radiotherapy dose of 45.0–50.4 Gy was used. In the study of Joye et al., patients received a total dose of 36–55.8 Gy [47]. In most studies, capecitabine or 5-FU was administered during CRT. Almost all patients underwent TME; only in two studies a small subset of patients was treated with a local excision [51,54]. Time between the end of CRT and surgery ranged from 4 to 12 weeks.

There were 14 (87.5%) model development studies (TRIPOD type 1b–3) [46,47,48,49,50,51,52,54,55,56,57,58,59,60]. None of the model development studies were externally validated in an independent study. Eleven studies performed internal validation using resampling methods (Type 1b) [46,47,48,49,50,51,52,54,55,56,57] and fives studies by randomly splitting the data (Type 2a) [48,56,58,59,60]. An intermediary (i.e., between internal and external) form of validation was performed in one study by nonrandomly splitting the data (Type 2b) [52]. Three development studies externally validated the model in the same study (Type 3) [52,57,58]. There were two (12.5%) model-validation-only studies (Type 4) [53,61]. The two validation-only studies validated models that were originally developed for a different outcome, which did not meet our inclusion criteria.

Table 3 shows the model and outcome characteristics of the selected studies. Supplementary Table S2 describes the final predictors per model in more detail. None of the studies looked at cCR or ncCR. Model outcomes were pCR (four studies) [49,52,57,58], pathological tumor response (10 studies) [46,48,51,53,54,55,56,59,60,61], or both (two studies) [47,50]. All studies dichotomized pathological tumor response into good versus poor response or synonyms of these terms. This dichotomous outcome is hereafter referred to as good response (GR). In all but one study [50], which used ypTN stage, dichotomization was conducted using one of the following tumor regression grade (TRG) systems [62,63,64,65,66,67]: American Joint Committee on Cancer (AJCC) [46,48,55,56], Mandard [53,54], Song [59,60], Dworak [61], Quirke [47], and Gastrointestinal Pathology Study Group of the Korean Society of Pathologists [51].

Nine studies used logistic regression as a modelling method during model development, sometimes in combination with other methods [46,47,49,50,51,56,57,60,61]. Other types of modelling methods were ridge regression (RR), support vector machine (SVM), lasso and elastic-net regularized generalized linear models (R package “glmnet”), partial least squares (PLS) analysis, repeated measure analysis of variance (RM-ANOVA), multilevel partial least squares discriminant analysis (ML-PLS-DA), multi-instance learning (MIL), convolutional neural network (CNN), and graph neural network (GNN).

### 3.3. PROBAST Risk of Bias Assessment

**Figure 2:**
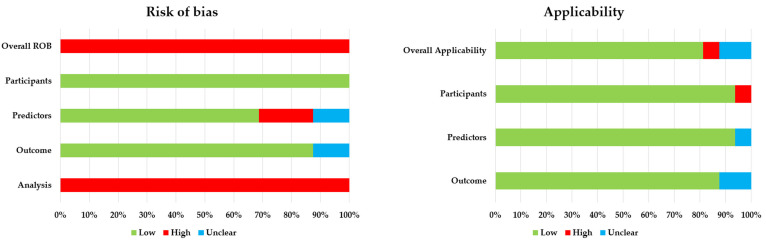
PROBAST risk of bias assessment. *ROB = risk of bias*.

Results of ROB assessment using PROBAST are shown in Table 4 and Figure 2. All studies were classified as having a high ROB, mainly because all studies had problems arising in the analysis domain. Reasons for a high ROB in the analysis domain were: 11 of 14 (79%) model development studies did not account for overfitting appropriately, mostly because resampling methods were not used for all model development procedures, including predictor selection. In 12 studies (75%), the low number of participants with the outcome (pCR/GR) compared to the number of candidate predictors could lead to a high ROB. Four of fourteen (29%) model development studies did not avoid predictor selection based on univariable analysis and three (19%) studies did not handle continuous and/or categorical predictors appropriately. Moreover, there was no information about handling of missing data and calibration in 13 (81%) and 11 (69%) studies, respectively, and the complete final model formula was not given in 13 (93%) of 14 model development studies. A high risk of bias in the predictors domain was seen in three studies because of reported heterogeneity in quality of biopsies.

There were only minor concerns with regard to applicability of the included studies: one study had high concerns and two studies had unclear concerns regarding applicability.

### 3.4. Model Results

#### 3.4.1. General

**Figure 3:**
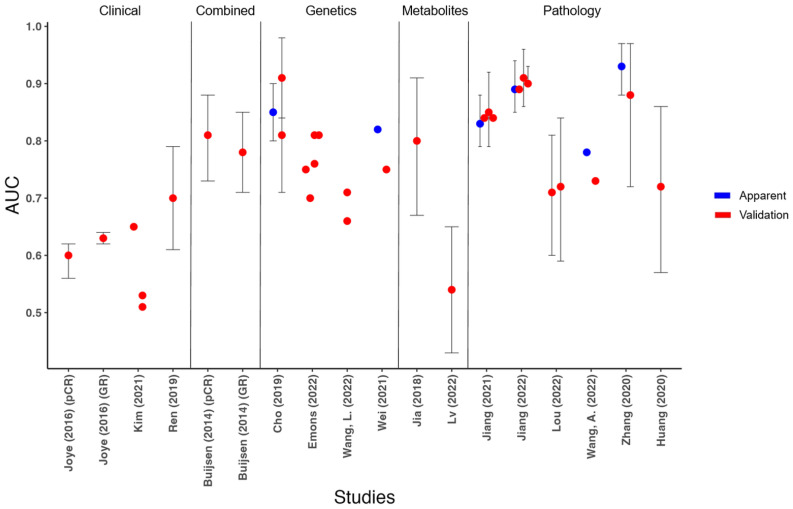
Discriminative performance with confidence intervals. *Y*-axis represents reported AUCs; *x*-axis represents included studies. Apparent performance was reported in six of fourteen model development studies [51,54,56,57,59,60]. The 95% confidence intervals were reported in 10 studies [46,47,49,50,51,55,56,57,58,60] *AUC* = *area under the curve*; *GR* = *good response*; *pCR* = *pathological complete response*.

AUCs of all studies ranged from poor to excellent (0.51–0.93) (Figure 3). AUCs were between 0.60 and 0.91 for the outcome pCR and 0.51 and 0.93 for the outcome GR. Apparent performance (discrimination of the model on the development cohort before internal validation) was reported in six of fourteen model development studies [51,54,56,57,59,60]. Five studies [49,54,56,57,60] described model calibration using the Hosmer–Lemeshow goodness-of-fit test, a calibration curve, or both. Calibration slopes and intercepts were not reported. Based on the predictors included in the final model, studies were grouped into the following five categories: clinical, combined (clinical, serological, and imaging), genetics, metabolites, and pathology. Besides the combined prediction model from Buijsen et al. [50], all models used predictors from a single category or combined those predictors only with standard clinical predictors.

#### 3.4.2. Clinical Prediction Models

Three studies developed a prediction model using clinical predictors only [47,48,49], with poor–moderate AUCs, ranging from 0.60 to 0.70. Joye et al. [47] developed and internally validated a model in one cohort (*n* = 620) for the outcomes pCR and good response (GR). Following backward predictor selection, the pCR model included six (age, American Society of Anesthesiologists (ASA) score, CEA, cN stage, gender, and hemoglobin (Hb)) and the GR model four (CEA, cT stage/mesorectal fascia (MRF), cN stage, and Hb) clinical predictors. Apparent AUCs were not reported. After bootstrapping, the pCR model had an AUC of 0.60 (range 0.56–0.62) and the GR model 0.63 (range 0.62–0.64). Kim et al. [48] evaluated a model on GR and randomly split the data into a development (*n* = 190), tuning (*n* = 41), and validation (*n* = 41) set. The clinical model included 10 predictors: age, alcohol, ASA, BMI, diabetes, distance from anal verge, gender, hypertension, smoking, and tumor grade. AUCs for the prediction of GR were 0.65 (mean ± std: 0.02), 0.53 (mean ± std 0.08), and 0.51 (mean ± std 0.08) in the training, tuning, and validation cohort. Kim et al. also developed models with additional serological predictors measured during CRT, of which the best model had slightly better discriminative performance than the pretreatment model. The study by Ren et al. [49] evaluated a model on pCR after CRT with mFOLFOX in 126 patients that included two predictors after forward predictor selection: MRF and tumor length. The apparent AUC was not reported; after bootstrapping the AUC was 0.70 (95% CI 0.61–0.79). Model calibration performance was evaluated with a calibration curve, which seemed to indicate miscalibration in the 0.2–0.4 predicted probability range.

#### 3.4.3. Combined Clinical, Serological and Imaging Prediction Model

Only one study (Buijsen et al.) [50] combined pretreatment predictors of more than two different categories in a model. Four clinical (CEA, cT stage, cN stage, and tumor length), three serological (interleukin (IL)-6, IL-8, and osteopontin), and one imaging (maximal standardized uptake value (SUVmax)) predictor(s) were selected. Data of 276 patients were used for development and internal validation of the model, predicting both pCR and GR. Apparent AUCs were not reported; bootstrapped AUCs for pCR and GR were good and moderate: 0.81 (95% CI 0.73–0.88) and 0.78 (95% CI 0.71–0.85), respectively.

#### 3.4.4. Genetics Prediction Models

Four models evaluated gene-based prediction models [51,52,53,54]. AUCs ranged widely, from poor to excellent (range 0.66–0.91). None of the genes were used in more than one model. The studies had small sample sizes (*n* = 24–184); only two studies used a cohort that consisted of more than 100 patients. Cho et al. [51] developed an eight-gene mRNA radio-response prediction index for GR using stepwise predictor selection in a cohort that included 184 patients, with an apparent AUC of 0.85 (95% CI 0.80–0.90) and AUCs after cross-validation that ranged from 0.81 (95% CI 0.71–0.91) to 0.91 (95% CI 0.84–0.98). Emons et al. [52] developed a 21-transcript signature for pCR prediction in a cohort of 64 patients using hill-climbing predictor selection. The apparent AUC was not reported. Several cohorts (*n* = 14–161) were used for internal and external validation, with AUCs between 0.70 and 0.81. The transient receptor potential channels (TRPC) score of Wang et al. [53], which reflects the expression of eight TRPG related genes, was developed using patients with colorectal cancer for the outcome overall survival. However, in the same article, the score was externally validated in two separate rectal cancer cohorts (*n* = 80 and *n* = 85) for the outcome GR, with AUCs of 0.66 and 0.71, respectively. In the study by Wei et al. [54], four immune-related differentially expressed genes were selected using least absolute shrinkage and selection operator (LASSO) regression and combined with two clinical predictors (age and gender) in a prediction nomogram. The analyzed cohort consisted of 59 patients that received chemotherapy as either 5-FU or capecitabine with or without oxaliplatin. The apparent AUC for GR was 0.82 and after bootstrapping 0.75. The graphically displayed calibration curve seemed to demonstrate slight miscalibration, especially in the higher predicted probability ranges.

#### 3.4.5. Metabolites Prediction Models

Two studies evaluated metabolite-based models, Jia et al. (2018) and Lv et al. (2022) [46,55]. Both cohorts (*n* = 105 and *n* = 106, respectively) were from the same hospital which treated patients with one additional consolidation cycle of chemotherapy after CRT. The studies used the same methods for metabolite analysis but included different metabolites in the final models. Both used the outcome GR. Apparent AUCs were not reported. The 15-metabolite panel from Jia et al. [55] had a good AUC of 0.80 (95% CI 0.67–0.91) after bootstrapping and the eight-metabolite panel from Lv et al. [46] had a poor AUC of 0.54 (95% CI 0.43–0.65) after cross-validation. Lv et al. measured and evaluated metabolites at multiple time points; the last measurement was within two days before surgery. The use of multiple measurements improved the cross-validated discriminative performance slightly.

#### 3.4.6. Pathology Prediction Models

There were six pathology-based prediction models [56,57,58,59,60,61]. AUCs were between 0.71 (good) and 0.91 (excellent) [56,57,58,59,60]. Jiang et al. published two collagen feature models, in 2021 [56] on GR and 2022 on pCR [57]. Both studies used data from patients in the same three hospitals, collected between 2010 and 2018. The collagen features were analyzed in the same manner using multiphoton images. One collagen feature was used in both models. In the study published in 2021, data were randomly split in a development (*n* = 299) and validation (*n* = 129) cohort. The authors first combined three collagen features in a Collagen Features Support Vector Machine (CFs-SVM) classifier, after which the CFs-SVM classifier was combined with three clinical predictors (CEA, cT stage, and tumor differentiation) in a nomogram for GR. Without the addition of clinical predictors, the CFs-SVM classifier had an AUC of 0.78 (95% CI 0.72–0.83) in the development and 0.77 (95% CI 0.68–0.85) in the validation cohort. The apparent AUC of the combined nomogram was 0.83 (95% CI 0.79–0.88). The nomogram was validated in the development and validation cohorts with AUCs of 0.84 and 0.85 (95% CI 0.79–0.92). Then, the nomogram was validated in a cohort that used all patients, from the development as well as the validation cohort, with an AUC of 0.84. Calibration curves were good and the Hosmer–Lemeshow (H-L) goodness-of-fit test results, with *p*-values of 0.096 (development) and 0.496 (validation), suggested no miscalibration. The dataset of the study published in 2022 consisted of a development (*n* = 353) and external validation (*n* = 163) cohort. The pCR model combined four collagen features (selected with LASSO regression) with four clinical predictors (CEA, cT stage, tumor differentiation, and tumor dimension (length)). The collagen signature, before combination with the clinical predictors, had an AUC of 0.84 (95% CI 0.79–0.90) in the development and 0.84 (95% CI 0.75–0.92) in the validation cohort. The apparent AUC of the final model that included clinical predictors was 0.89 (95% CI 0.85–0.94), with validated AUCs between 0.89 and 0.91 (95% CI 0.86–0.96). Calibration curves showed good calibration and there was no suggestion for miscalibration based on the *p*-values from the H-L goodness-of-fit test, which were 0.314 in the development and 0.670 in the validation cohort.

Three models evaluated the predictive value of pathological whole slide images (WSIs). Lou et al. [58] developed a deep learning model with 666, 117, and 102 participants in the training, testing, and external validation cohort, respectively. The apparent AUC was not reported. AUCs for pCR in the testing and external validation cohorts were 0.71 (95% CI 0.60–0.81) and 0.72 (95% CI 0.59–0.84). Wang et al. published a digital-pathology-based deep learning model [59]. Patients in the development (*n* = 55) and randomly split (*n* = 14) cohorts were treated with induction chemotherapy (FOLFOX or CAPOX) and CRT. The evaluated model used a maximum of 500 samples from collected WSIs, which were processed and analyzed using a convolutional neural network and a graph neural network. The model showed an apparent AUC of 0.78 and an AUC of 0.73 in the randomly split cohort. Zhang et al. [60] developed a pathology signature with 17 predictors, selected from WSIs using LASSO, trained for the outcome GR. A total of 151 patients were randomly split in a development (*n* = 120) and validation (*n* = 31) dataset. A subset of 78 (52%) patients received two additional cycles of consolidation chemotherapy (capecitabine or 5-FU/leucovorin) after CRT. It was unclear how many patients with GR received consolidation chemotherapy. The apparent AUC was 0.93 (95% CI 0.88–0.97) and the AUC of the nonrandomly split cohort 0.88 (95% CI 0.72–0.97). Calibration curves showed slight miscalibration in the lower and higher predicted probability ranges, although this was not seen in the *p*-values of the H-L goodness-of-fit test (0.332 and 0.213).

One study (Huang et al.) [61] externally validated the immunoscore, a prediction model that uses tumor-infiltrating lymphocytes (TILs). They evaluated the score using CD3+ and CD8+ TILs in a small cohort of rectal cancer patients (*n* = 55). The AUC for GR was 0.72. It is important to note that the immunoscore was originally not developed to predict the response to neoadjuvant therapy but, rather, to evaluate prognostic outcomes in colorectal patients who underwent surgery without neoadjuvant therapy [68,69].

## 4. Discussion

In this systematic review we summarized and critically appraised 16 validated pretreatment prediction models for response to neoadjuvant therapy in patients with rectal cancer. The models were grouped into five categories and included several promising predictors, with some models showing encouraging predictive potential. However, calibration outcomes were only reported in five studies and PROBAST indicated that all studies had a high risk of bias (ROB), mainly in the analysis domain. In addition, the majority of studies used small sample sizes, and external validation in independent studies was lacking. Based on these findings, we propose some recommendations for future research.

The two collagen feature-based models from Jiang et al. [56,57] showed the most promising results of all studies. The models reported good to excellent discriminative performance (AUCs 0.83–0.91) with relatively small confidence intervals, good calibration, and consistency across cohorts. The discriminative performance of both collagen-only signatures improved with the addition of clinical predictors, suggesting that the collagen features may be complementary to standard clinical predictors, rather than explain similar aspects of the tumor. Furthermore, multiphoton imaging for collagen feature analysis offers some advantages, such as rapid analysis using routinely collected pretreatment biopsies and a quick learning curve for newly trained pathologists [56,57,70,71]. In addition, comparable pretreatment collagen feature models showed good and excellent discrimination for prognostic outcomes in colon cancer (*n* = 882, disease-free survival/overall survival) and gastric cancer (*n* = 375, lymph node metastasis), respectively [71,72]. Thus, collagen feature-based prediction models are good potential candidates for further (external) validation, despite the high ROB of the included studies.

The pathological whole slide image (WSI)-based models [58,59,60], immunoscore [61], and metabolite-based models [46,55] reported poor to excellent discriminative performance (AUCs 0.54–0.93). Due to the heterogeneous results, small sample sizes, and high ROB, it is challenging to determine their exact predictive value. Consequently, we cannot confidently recommend these models for further validation. Nevertheless, the included predictors remain interesting for future research. Digital WSIs of pretreatment biopsies can be used to quantify pathological features [59,73]. WSI analysis has the potential to be easily incorporated in routine clinical practice, since standard rectal cancer biopsies can be used and the annotation workload for the pathologist is significantly reduced with new analysis and modelling methods [59,60,74]. Metabolomics is defined as the analysis of intermediate or end products of metabolic processes (metabolites) [75]. It is an easily obtained and minimally invasive method for response prediction, with serum as the preferred sample for analysis, but the literature about its role for the prediction of treatment response in patients with rectal cancer is scarce [76]. The immunoscore was originally developed [68] and later validated [69] for prognostic outcomes in colorectal patients undergoing surgery without neoadjuvant therapy. The score combines the densities of two tumor-infiltrating lymphocytes (TILs) populations at the center of the tumor and at the invasive margin. In addition to the included study from Huang et al. [61], El Sissy et al. [77] validated the pretreatment immunoscore in a cohort of 249 rectal cancer patients and showed a positive correlation of the immunoscore with tumor response and significantly higher pCR rates in patients with a high immunoscore. This study was excluded for data extraction and ROB assessment in the present review because tumors other than adenocarcinoma were included. The immunoscore has strong interobserver reproducibility [69] but the exact value of the immunoscore for pretreatment response prediction remains to be determined.

The four gene-based models [51,52,53,54] showed widely varying discriminative performances, ranging from poor to excellent (AUCs 0.66–0.91). Small sample sizes were used, a common issue in rectal cancer gene-based prediction model studies [30,31,78,79]. A second frequently occurring problem is the lack of overlap between genes in different studies [51,80]. None of the genes from the models included in this review were used in more than one model. Moreover, Cho et al. [51] compared their eight-mRNA gene model with 11 other gene expression studies and found that only one of their eight mRNA genes showed predictive value in other studies. Furthermore, a systematic review by Izzotti et al. identified 674 mRNAs and 77 microRNAs with potential predictive value for response to neoadjuvant therapy in colorectal cancer patients and only 19 mRNAs and 6 microRNAs were differentially expressed in more than one study [81]. The results of the gene-based models included in this review confirm the need for larger sample sizes and genes that show consistent predictive value across multiple prediction models.

We did not include radiomics-based and image-based deep learning models in this review because of the already available recent reviews. Two systematic reviews evaluated the predictive value of pretreatment radiomics-based models. Staal et al. evaluated 24 studies [32] and Di Re et al. 9 studies [34]. AUCs ranged from 0.47 to 0.99. A meta-analysis from 2022 by Jia et al. [33] evaluated 21 MRI radiomics-based models and image-based deep learning models. The pooled AUC of the validation cohorts was excellent (0.92 with 95% CI: 0.88–0.93), although a small number of studies also included models with both pre- and post-treatment predictors. As shown by these systematic reviews, the number of radiomics-based and image-based deep learning models is high and good–excellent AUCs were often reported. However, the models suffer from similar problems as those included in the current review, such as a high ROB, reproducibility issues, and a lack of external validation [32,34]. Consequently, radiomics-based models and image-based deep learning models are not ready for clinical practice, pending external validation in large multicenter prospective trials.

PROBAST indicated a high ROB in all studies, mainly because all studies had a high ROB in the analysis domain. We obtained these results despite only selecting studies that reported some form of internal or external validation. The results are in line with other systematic reviews that use PROBAST, as is shown in a recent meta-review of 50 systematic reviews that used PROBAST. Included studies were often considered to have a high or unclear ROB, mostly because of problems in the analysis domain [82]. The negative effect of a high ROB is quantified by Venema et al. [83], who rated 102 prediction models with a shortened form that included six PROBAST items. The median change in discrimination (AUC) between development and validation cohorts was significantly higher in high ROB models (−11.7%) compared to low ROB models (−0.9%). Large sample sizes that include a sufficient number of participants with the outcome are critical to minimize ROB. Twelve of sixteen (75%) included studies had a low number of participants with the outcome (pCR/GR) compared to the number of candidate predictors. A sample size calculation can be helpful to identify the maximum number of candidate predictors or to identify a dataset that is too small for a particular research question [84]. A problem such as the effect of not properly accounting for overfitting, which was seen in most included development studies, might even be negligible if a dataset is used with a sufficient number of participants with the outcome [83]. Unfortunately, even in large clinical rectal cancer trials, it is difficult to include a reasonable number of patients with pCR or GR, let alone patients with a (near) cCR. Alternatively, data collection in prospective observational cohort studies may improve sample size numbers.

Our study has some limitations. We could have missed interesting studies by excluding those that included patients with mucinous, signet-ring cell, or neuroendocrine tumors. However, these tumors may respond differently to neoadjuvant therapy than standard adenocarcinomas because of distinct tumor biology [85,86,87,88], which would have led to more heterogeneous results. Moreover, by excluding radiomics-based and image-based deep learning models, we might have missed potential studies that combine these models with predictors from other categories. Even so, we believe that most relevant models were included in the reviews of Di Re, Staal, and Jia [32,33,34], since these reviews also included models that added predictors from different categories.

The substantial ROB of selected studies in this review prevents the implementation of these models in a clinical setting. However, our findings indicate that some pretreatment prediction models, in particular the collagen-feature models and perhaps the genetics-, metabolites-, and pathology-based models, could have important clinical value in the future. Several recommendations can be given to guide future research on the additional clinical value of these models. The models should be further developed and externally validated in independent cohorts with a sufficient sample size, and appropriate statistical plans should be designed to minimize bias. Furthermore, easily obtainable predictors should be used that are measured with standardized methods to allow reproducibility of the results. In addition, the models need to be validated for organ preservation outcomes instead of postoperative outcomes given the conflicting results that exist regarding the concordance between cCR and pCR [9,89,90]. Finally, to improve generalizability, investigated patient populations should also include patients with early tumor stages and neoadjuvant treatment regimens other than CRT. These steps might enable us to select the most promising models that should be evaluated in the clinic and discard those models that are not worthy of further investigation.

## 5. Conclusions

This systematic review aimed to summarize and critically appraise validated pretreatment prediction models for response to neoadjuvant therapy in patients with rectal cancer and provide evidence-based recommendations for future research. Results were heterogeneous, all studies were considered to have a high risk of bias, and external validation in independent studies was lacking. Despite these limitations, several promising predictors were identified, and some models demonstrated encouraging predictive potential, in particular the collagen feature-based models. These studies could form the basis for future research, which should focus on the reduction of bias, evaluate reproducible predictors, and use outcomes specifically tailored to organ preservation. Furthermore, it is crucial that these models undergo external validation in independent studies in order to reach clinical value.

## Figures and Tables

**Table 1:** Types of prediction model studies according to the TRIPOD (transparent reporting of a multivariable prediction model for individual prognosis or diagnosis) statement [39].

Study Type	TRIPOD Type	Development	Type of Validation	Explanation
**Development studies**				
Development only	1a	Yes	No	Development and evaluation using the apparent performance.
**Development and validation studies**				
Development and validation using resampling	1b	Yes	Internal	Development and evaluation using resampling methods (e.g., cross-validation or bootstrapping). The term “internal validation” commonly refers to resampling methods.
Development and validation using random split	2a	Yes	Internal	Random split of data into a development and validation cohort.
Development and validation using nonrandom split	2b	Yes	Intermediary	Nonrandom split (e.g., location or time) of data into a development and validation cohort. Type 2b studies are considered as intermediary between internal and external validation.
Development and validation using separate data	3	Yes	External	Development using one cohort and validation in a separate cohort (e.g., from a different study).
**Validation studies**				
Validation only	4	No	External	Validation of an existing (published) prediction model in a separate cohort.

**Table 2:** Cohort characteristics of the 16 included studies.

Study, [Ref.]	Model Development (Yes/No)	Validation Cohort(s) (No. of Cohorts and TRIPOD Category)	Cohort Design	*n*	*n* with Outcome (%)	Stage	NAT	Surgery
**Clinical**								
Joye (2016), [47]	Yes	1. 1b	R/S	620	pCR: 120 (19) GR: 170 (27)	cT1-4N0-2	CRT	TME
Kim (2021), [48]	Yes	1. 1b	R/S	190	83 (43)	LARC ^¥¥^	CRT	TME
		2. 2a	R/S	41	19 (47)	LARC	CRT	TME
		3. 2a	R/S	41	19 (47)	LARC	CRT	TME
Ren (2019), [49]	Yes	1. 1b	R/S	126	45 (36)	cT2-4N0-+	CRT	TME
**Clinical, serological and radiological**								
Buijsen (2014), [50]	Yes	1. 1b	P/S	276	pCR: 57 (21) GR: 130 (47)	cT3-4N0-2	CRT	TME
**Genetics**								
Cho (2019), [51]	Yes	1. 1b	R/S	184	102 (55)	cT3-4N0-+	CRT	TME (178) + LE (6)
Emons (2022), [52]	Yes	1. 1b	P/M	64	32 (50)	II-III	CRT	TME
		2. 1b/2b ^§^	P/M	161	32 (20)	II-III	CRT	TME
		3. 2b	P/M	14	4 (28)	II-III	CRT	TME
		4. 3	R/S	38	8 (21)	cT3-4N0-2	CRT	TME
		5. 3	P/M	24	4 (16)	II-III	CRT	TME
Wang, L. (2022), [53]	No	1. 4	R/S	80	35 (44)	cT3-4N0-2	CRT	TME
		2. 4	R/S	85	45 (53)	II-III	Unknown	Unknown
Wei (2021), [54]	Yes	1. 1b	R/S	59	27 (46)	cT3-4N0-2	CRT	TME (52) + LE (7)
**Metabolites**								
Jia (2018), [55]	Yes	1. 1b	P/S	105	56 (53)	cT3-4N0-2	CRT + Con.	TME
Lv (2022), [46]	Yes	1. 1b	P/S	106	56 (53)	cT2-4N0-2	CRT + Con.	TME
**Pathology**								
Jiang (2021), [56]	Yes	1. 1b	R/M	299	163 (55)	cT3-4N0-+	CRT	TME
		2. 2a	R/M	129	70 (54)	cT3-4N0-+	CRT	TME
		3. Cohort 1 + 2 ^‡^	R/M	428	233 (54)	cT3-4N0-+	CRT	TME
Jiang (2022), [57]	Yes	1. 1b	R/M	353	76 (22)	cT3-4N0-+	CRT	TME
		2. 3	R/S	163	37 (23)	cT3-4N0-+	CRT	TME
		3. Cohort 1 + 2 ^‡^	R/M	516	113 (22)	cT3-4N0-+	CRT	TME
Lou (2022), [58]	Yes	1. –	R/M	666	171 (26)	cT3-4N0-2	CRT	TME
		2. 2a	R/M	117	30 (26)	cT3-4N0-2	CRT	TME
		3. 3	R/M	102	24 (24)	cT3-4N0-2	CRT	TME
Wang, A. (2022), [59]	Yes	1. -	R/S	55	21 (38)	cT2-4N0-2	Ind. + CRT	TME
		2. 2a	R/S	14	5 (37)	cT2-4N0-2	Ind. + CRT	TME
Zhang (2020), [60]	Yes	1. -	R/S	120	59 (49)	cT2-4N0-2	CRT + Con.	Unknown
		2. 2a	R/S	31	20 (65)	cT2-4N0-2	CRT + Con.	Unknown
Huang (2020), [61]	No	1. 4	R/S	55	34 (62)	cT3-4N0-+	CRT	TME

Con. = consolidation chemotherapy; CRT = chemoradiotherapy; GR = good response; Ind. = induction chemotherapy; LARC = locally advanced rectal cancer; LE = local excision; M = multicenter; *n* = number of patients; NAT = neoadjuvant therapy; P = prospective; pCR = pathological complete response; R = retrospective; Ref. = reference; S = single-center; TME = total mesorectal excision; TRIPOD = transparent reporting of a multivariable prediction model for individual prognosis or diagnosis. ¥¥ = more detailed tumor stage was not reported; § = the 64 patients of cohort 1 were also included in cohort 2; ‡ = cohort that used all patients, from the development as well as the validation cohort.

**Table 3:** Model characteristics of the 16 included studies.

Study, [Ref.]	Model with Final Predictors (No. of Predictors)	Method	Outcome	Apparent AUC	AUC of Validation Cohort(s) (No. of Cohorts and TRIPOD Category)
**Clinical**					
Joye (2016), [47]	- Model 1 (pCR): age, ASA score, CEA, cN stage, gender and Hb. (6) - Model 2 (GR): CEA, cT stage/MRF, cN stage and Hb. (4)	LR	pCR GR	pCR. Unknown GR. Unknown	1. 1b. (pCR) 0.60 (range 0.56–0.62) 1. 1b. (GR) 0.63 (range 0.62–0.64)
Kim (2021), [48]	- Age, alcohol, ASA, BMI, diabetes, distance from anal verge, gender, hypertension, smoking and tumor grade. (10)	RR	GR	Unknown	1. 1b. 0.65 (mean ± std: 0.02) 2. 2a. 0.53 (mean ± std 0.08) 3. 2a. 0.51 (mean ± std 0.08)
Ren (2019), [49]	- MRF and tumor length. (2)	LR	pCR	Unknown	1. 1b. 0.70 (95% CI 0.61–0.79)
**Clinical, serological and imaging**					
Buijsen (2014), [50]	- CEA, cT stage, cN stage and tumor length, IL-6, IL-8, osteopontin, SUVmax. (8)	LR	pCR GR	pCR. Unknown GR. Unknown	1. 1b. (pCR) 0.81 (95% CI 0.73–0.88) 1. 1b. (GR) 0.78 (95% CI 0.71–0.85)
**Genetics**					
Cho (2019), [51]	Radio-response prediction index - mRNAs. (8)	LR	GR	0.85 (95% CI 0.80–0.90)	1. 1b. 0.81 (95% CI 0.71–0.91)–0.91 (95% CI: 0.84–0.98) ^¥¥^
Emons (2022), [52]	21-transcript signature - Transcripts. (21)	SVM	pCR	Unknown	1. 1b. 0.75 2. 1b/2b ^§^. 0.81 3. 2b. 0.70 4. 3. 0.76 5. 3. 0.81
Wang, L. (2022), [53]	Transient receptor potential channels (TRPC) score - TRPC-related genes. (8)	R package “glmnet”	GR	NA	1. 4. 0.66 2. 4. 0.71
Wei (2021), [54]	Response-Related Prediction Nomogram - IRDEGs, age and gender. (6)	R package “glmnet”	GR	0.82	1. 1b. 0.75
**Metabolites**					
Jia (2018), [55]	Metabolite panel - Metabolites. (15)	PLS	GR	Unknown	1. 1b. 0.80 (95% CI 0.67– 0.91)
Lv (2022), [46]	Metabolite panel - Metabolites. (8)	RM-ANOVA, ML-PLS-DA and LR	GR	Unknown	1. 1b. 0.54 (95% CI 0.43–0.65)
**Pathology**					
Jiang (2021), [56]	Collagen feature nomogram - CFs-SVM classifier, CEA, cT stage, and tumor differentiation. (6)	SVM and LR	GR	0.83 (95% CI 0.79–0.88)	1. 1b. 0.84 2. 2a. 0.85 (95% CI 0.79–0.92) 3. Cohort 1 + 2 ^‡^. 0.84
Jiang (2022), [57]	Collagen feature nomogram - Collagen signature, CEA, cT stage, tumor differentiation, and tumor dimension (length). (8)	LR	pCR	0.89 (95% CI 0.85–0.94)	1. 1b. 0.89 2. 3. 0.91 (95% CI 0.86–0.96) 3. Cohort 1 + 2 ^‡^. 0.90 (95% CI 0.90–0.93)
Lou (2022), [58]	Deep pathological complete response model - WSIs. (NA)	MIL	pCR	Unknown	2. 2a. 0.71 (95% CI 0.60–0.81) 3. 3. 0.72 (95% CI 0.59–0.84)
Wang, A. (2022), [59]	Digital-pathology-based deep learning model - WSIs. (NA)	CNN and GNN	GR	0.78	2. 2a. 0.73
Zhang (2020), [60]	Pathology signature - WSIs. (17)	SVM and LR	GR	0.93 (95% CI 0.88–0.97)	2. 2a. 0.88 (95% CI 0.72–0.97)
Huang (2020), [61]	Immunoscore - TILs. (4)	LR	GR	NA	1. 4. 0.72 (95% CI 0.57–0.86)

95% CI = 95% confidence interval; ASA = American Society of Anesthesiologists score; AUC = area under the curve; CEA = carcinoembryonic antigen; CFs-SVM classifier = Collagen Features Support Vector Machine classifier; CNN = convolutional neural network; glmnet = lasso and elastic-net regularized generalized linear models; GNN = graph neural network; GR = good response; Hb = hemoglobin; IL = interleukin; IRDEGs = immune-related differentially expressed genes; MIL = multi-instance learning; ML-PLS-DA = multilevel partial least squares discriminant analysis; MRF = mesorectal fascia; mRNA = messenger RNA; *n* = number of predictors in final model; NA = not applicable; LR = logistic regression; pCR = pathological complete response; PLS = partial least squares; Ref. = reference; RM-ANOVA = repeated measure analysis of variance; RR = ridge regression; SUVmax = maximal standardized uptake value; SVM = support vector machine; TILs = tumor-infiltrating lymphocytes; TRIPOD = transparent reporting of a multivariable prediction model for individual prognosis or diagnosis; TRPC = transient receptor potential channels; U = unknown; WSIs = whole slide images. ¥¥ = range of cross-validated AUCs; § = the 64 patients of cohort 1 were also included in cohort 2; ‡ = cohort that used all patients, from the development as well as the validation cohort.

**Table 4:** PROBAST risk of bias assessment.

Study	Type	ROB				Applicability			Overall	
		Participants	Predictors	Outcome	Analysis	Participants	Predictors	Outcome	ROB	Applicability
**Clinical**										
Joye (2016), [47]	D	Low	Low	Low	High	Low	Low	Low	High	Low
Kim (2021), [48]	D	Low	Low	Low	High	Low	Unclear	Low	High	Unclear
Ren (2019), [49]	D	Low	Low	Low	High	Low	Low	Low	High	Low
**Clinical, serological and radiological**										
Buijsen (2014), [50]	D	Low	Low	Low	High	Low	Low	Low	High	Low
**Genetics**										
Cho (2019), [51]	D	Low	Low	Low	High	Low	Low	Low	High	Low
Emons (2022), [52]	D + V	Low	Low	Low	High	Low	Low	Low	High	Low
Wang, L. (2022), [53]	V	Low	High	Unclear	High	High	Low	Unclear	High	High
Wei (2021), [54]	D	Low	Low	Low	High	Low	Low	Low	High	Low
**Metabolites**										
Jia (2018), [55]	D	Low	Unclear	Low	High	Low	Low	Low	High	Low
Lv (2022), [46]	D	Low	Unclear	Low	High	Low	Low	Low	High	Low
**Pathology**										
Jiang (2021), [56]	D	Low	Low	Low	High	Low	Low	Low	High	Low
Jiang (2022), [57]	D + V	Low	Low	Low	High	Low	Low	Low	High	Low
Lou (2022), [58]	D + V	Low	Low	Low	High	Low	Low	Low	High	Low
Wang, A. (2022), [59]	D	Low	High	Unclear	High	Low	Low	Unclear	High	Unclear
Zhang (2020), [60]	D	Low	High	Low	High	Low	Low	Low	High	Low
Huang (2020), [61]	V	Low	Low	Low	High	Low	Low	Low	High	Low

D = development; ROB = risk of bias; V = validation.

## Supplementary Materials

The following supporting information can be downloaded at: https://www.mdpi.com/article/10.3390/cancers15153945/s1, Table S1: Complete search. Table S2: Overview of final predictors per model.
