# Supplementary material for: Validated Pretreatment Prediction Models for Response to Neoadjuvant Therapy in Patients with Rectal Cancer: A Systematic Review and Critical Appraisal
Source: Cancers (Basel). 2023 Aug 3;15(15):3945. doi: 10.3390/cancers15153945 (PMC10417363; doi:10.3390/cancers15153945)
Supplement: Supplementary file 1 [file cancers-15-03945-s001.zip › cancers-2481230-supplementary.pdf]

Index

- Table S1. Complete search
- Table S2. Overview of final predictors per model

Supplementary Table S1. Complete search

**Ovid MEDLINE**
**Query**
"rectal neoplasms"/
(rect* adj5 (carcinoma* or adenocarcinoma* or cancer* or neoplas* or malignan* or tumor* or tumour*)).ti,ab,kf.
or/1-2 [Rectal cancer]
respon*.ti,ab,kf.
pCR.ti,ab,kf.
cCR.ti,ab,kf.
"regression grade*".ti,ab,kf.
"regression score*".ti,ab,kf.
"tumor regression".ti,ab,kf.
"tumour regression".ti,ab,kf.
"tumor stag*".ti,ab,kf.
"tumour stag*".ti,ab,kf.
TRG.ti,ab,kf.
or/4-13 [response]
"neoadjuvant therapy"/
neoadjuvant.ti,ab,kf.
NAT.ti,ab,kf.
*"preoperative care"/
preoperative.ti,ab,kf.
"pre-operative".ti,ab,kf.
or/15-20 [neoadjuvant]
exp chemoradiotherapy/
"Chemotherapy, Adjuvant"/ and "radiotherapy, adjuvant"/ and 19940101:20111231.(dt).
chemoradiotherap*.ti,ab,kf.
chemoradiation.ti,ab,kf.
(chemo adj3 radiotherap*).ti,ab,kf.
"chemo-radiation".ti,ab,kf.
radiochemotherap*.ti,ab,kf.
(radio adj3 chemotherap*).ti,ab,kf.
"radiation-chemotherap*".ti,ab,kf.
CRT.ti,ab,kf.
nCRT.ti,ab,kf.
("short-course" adj3 (radiotherap* or radiation)).ti,ab,kf.
("long-course" adj3 (radiotherap* or radiation)).ti,ab,kf.
SCRT.ti,ab,kf.
SCPRT.ti,ab,kf.
("total neoadjuvant" adj2 (therap* or treatment*)).ti,ab,kf.
TNT.ti,ab,kf.
or/22-38 [crt/scrt/tnt]
3 and 14 and (21 or 39) [Rectal Cancer AND Response AND (Neoadjuvant therapy OR CRT/SCRT/TNT)]
**Embase.com**
**Query**
'rectum cancer'/de
'rectum carcinoma'/de

(rect* NEAR/5 (carcinoma* OR adenocarcinoma* OR cancer* OR neoplas* OR malignan* OR tumor* OR tumour*)):ti,ab,kw
#1 OR #2 OR #3
respon*:ti,ab,kw
pcr:ti,ab,kw
ccr:ti,ab,kw
'regression grade':ti,ab,kw
'regression score':ti,ab,kw
'tumor regression':ti,ab,kw
'tumour regression':ti,ab,kw
'tumor stag*':ti,ab,kw
'tumour stag*':ti,ab,kw
trg:ti,ab,kw
#5 OR #6 OR #7 OR #8 OR #9 OR #10 OR #11 OR #12 OR #13 OR #14
'neoadjuvant therapy'/exp
neoadjuvant:ti,ab,kw
nat:ti,ab,kw
'preoperative care'/mj
preoperative:ti,ab,kw
'pre-operative':ti,ab,kw
#16 OR #17 OR #18 OR #19 OR #20 OR #21
'chemoradiotherapy'/exp
chemoradiotherap*:ti,ab,kw
chemoradiation:ti,ab,kw
(chemo NEAR/3 radiotherap*):ti,ab,kw
'chemo-radiation':ti,ab,kw
radiochemotherap*:ti,ab,kw
(radio NEAR/3 chemotherap*):ti,ab,kw
'radiation-chemotherap*':ti,ab,kw
crt:ti,ab,kw
ncrt:ti,ab,kw
('short-course' NEAR/3 (radiotherap* OR radiation)):ti,ab,kw
('long-course' NEAR/3 (radiotherap* OR radiation)):ti,ab,kw
scrt:ti,ab,kw
scprt:ti,ab,kw
('total neoadjuvant' NEAR/2 (therap* OR treatment*)):ti,ab,kw
tnt:ti,ab,kw
#23 OR #24 OR #25 OR #26 OR #27 OR #28 OR #29 OR #30 OR #31 OR #32 OR #33 OR #34 OR #35 OR #36 OR #37 OR #38
#4 AND #15 AND (#22 OR #39)
#4 AND #15 AND (#22 OR #39) NOT ('conference abstract'/it OR 'conference paper'/it OR 'conference review'/it)
#4 AND #15 AND (#22 OR #39) AND ('conference abstract'/it OR 'conference paper'/it OR 'conference review'/it)
**Scopus.com**
**Query**

```
(TITLE-ABS-KEY((rect*) W/5 (carcinoma* OR adenocarcinoma* OR cancer* OR neoplas* OR malignan* OR tumor* OR tumour*)))
AND
(TITLE-ABS-KEY(respon* OR {pCR} OR {cCR} OR {regression grade*} OR {regression score*} OR {tumor regression} OR {tumour regression} OR "tumor stag*" OR "tumour stag*" OR {TRG}))
AND
((TITLE-ABS-KEY({neoadjuvant} OR {NAT} OR {preoperative} OR {pre-operative})) OR ((TITLE-ABS-KEY(chemoradiotherap* OR {chemoradiation} OR {chemo-radiation} OR radiochemotherap* OR {radiation-chemotherap*} OR {CRT} OR {nCRT} OR {SCRT} OR {SCPRT} OR {TNT}) OR TITLE-ABS-KEY(chemo W/3 radiotherap*) OR TITLE-ABS-KEY(radio W/3 chemotherap*) OR TITLE-ABS-KEY("short-course" W/3 (radiotherap* OR radiation)) OR TITLE-ABS-KEY("long-course" W/3 (radiotherap* OR radiation)) OR TITLE-ABS-KEY("total neoadjuvant" W/2 (therap* OR treatment*)))))
AND (EXCLUDE (DOCTYPE , "cp") OR EXCLUDE (DOCTYPE , "cr"))
```

Supplementary Table S2. Overview of final predictors per model

Study	Final predictors
**Clinical**	
Joye (2016)	- Model 1 (pCR): age, ASA, CEA, cN stage, gender and Hb. (6)<br>- Model 2 (GR): CEA, cT stage/ MRF, cN stage and Hb. (4)
Kim (2021)	Age, alcohol, ASA, BMI, diabetes, distance from anal verge, gender, hypertension, smoking and tumour grade. (10)
Ren (2019)	MRF and tumour length (2)
**Clinical, serological and radiological**	
Buijsen (2014)	<u>Total: 8</u><br>- Clinical (4): CEA, cT stage, cN stage and tumour length<br>- Serological (3): IL-6, IL-8 and osteopontin<br>- Radiological (1): SUVmax
**Genetics**	
Cho (2019)	mRNA (8): FZD9, HRAS, ITGA7, MECOM, MMP3, NKD1, PIK3CD and PRKCB
Emons (2022)	Transcripts (21): ASPM, BLM, BRCA1, CASC5, CCNB1, CENPL, CFAP61, CGREF1, CSPP1, CXCL10, DNAAF3, FANCM, FZD10, HOMER1, MCM5, SHTN1, STARD3, TMPO, TNPO3, TSNAX and XPO1.
Wang, L. (2022)	TRPC-related genes (8): CD177, CLDN23, FJX1, MTOR4, PCOLCE2, PPARGC1A, TIMP1, and UCN.
Wei (2021)	<u>Total: 6</u><br>- IRDEGs (4): ANGPT1, FLT3, GHR and HSPA2.<br>- Clinical (2): age and gender.
**Metabolites**	
Jia (2018)	Metabolites (15): M100T44 (d-Valerolactam), M127T112 (4-Imidazoleacetic acid), M156T112 (N-Methylethanolamine phosphate), M170T372 (3-Methylhistidine), M229T311_1 (Dimethylglycine), M278T269 (Dillapional), M412T212 (PC (9:0)), M501T111 (Oleanolic Acid Acetate), M650T136 (PC(25:1(OH))), M678T134 (PC(27:1(OH))), M692T255 (Ganglioside GT1b (d18:0/14:0)), M696T156 (SM(d18:2/14:0)), M805T45 (PC(16:0/18:1)), M817T255 (Unknown) and M833T255 (Unknown).
Lv (2022)	Metabolites (8): 1-Hexadecanoyl-sn-glycero-3-phosphocholine, acetylcholine, alpha-ketoisovaleric acid, betaine, glycerophosphocholine, hypoxanthine, L-Norleucine and N-Acetyl-L-alanine.
**Pathology**	
Jiang (2021)	<u>Total: 6</u><br>- CFs-SVM classifier (3): The classifier consists of 3 collagen structural features: collagen percentage area, crosslink density and mean of fiber straightness. The outcome of the CFs-SVM classifier was dichotomized and used as 1 predictor in the multivariable LR model.<br>- Clinical (3): CEA, cT stage and tumour differentiation.
Jiang (2022)	<u>Total: 8</u><br>- Collagen signature (4): the signature consists of 4 collagen features: collagen crosslink density, collagen orientation, collagen straightness and gabor_scale4_orientation3_mean. The outcome of the collagen signature was used as 1 predictor in the multivariable LR model.<br>- Clinical (4): CEA, cT stage, tumour differentiation and tumour dimension (length).
Lou (2022)	WSIs (NA): NA, deep learning model.

Wang, A. (2022)	WSIs (NA): NA, deep learning model.
Zhang (2020)	WSIs (17): NGTDM_Busyness, NGTDM_Contrast, NGTDM_Coarseness, GLCM_Energy, LBP7, LBP10, LBP12, LBP13, LBP14, LBP24, LBP26, LBP29, LBP30, Moment10, perceptual_Directionality, perceptual_LineLikeliness and prctile_25.
Huang (2020)	TILs (4): CD3+ (CT), CD3+ (IM), CD8+ (CT) and CD8+ (IM).

ASA = American Society of Anesthesiologists score; CEA = carcinoembryonic antigen; CFs-SVM classifier = Collagen Features Support Vector Machine classifier; CT = central region of the tumour; Hb = hemoglobin; IL = interleukin; IM = invasion margin; IRDEGs = immune-related differentially expressed genes; MRF = mesorectal fascia; mRNA = messenger-RNA; NA = not applicable; Ref. = reference; SUV = maximal standardized uptake value; SVM = support vector machine; TILs = tumour-infiltrating lymphocytes; TRPC = transient receptor potential channels; WSIs = whole slide images.
